# Leucine Supplementation Improves Skeletal Muscle Regeneration after Cryolesion in Rats

**DOI:** 10.1371/journal.pone.0085283

**Published:** 2014-01-08

**Authors:** Marcelo G. Pereira, Igor L. Baptista, Eduardo O. C. Carlassara, Anselmo S. Moriscot, Marcelo S. Aoki, Elen H. Miyabara

**Affiliations:** 1 Department of Anatomy, Institute of Biomedical Sciences, University of Sao Paulo, Sao Paulo, Brazil; 2 School of Arts, Sciences and Humanities, University of Sao Paulo, Sao Paulo, Brazil; Kaohsiung Chang Gung Memorial Hospital, Taiwan

## Abstract

This study was undertaken in order to provide further insight into the role of leucine supplementation in the skeletal muscle regeneration process, focusing on myofiber size and strength recovery. Young (2-month-old) rats were subjected or not to leucine supplementation (1.35 g/kg per day) started 3 days prior to cryolesion. Then, soleus muscles were cryolesioned and continued receiving leucine supplementation until 1, 3 and 10 days later. Soleus muscles from leucine-supplemented animals displayed an increase in myofiber size and a reduction in collagen type III expression on post-cryolesion day 10. Leucine was also effective in reducing FOXO3a activation and ubiquitinated protein accumulation in muscles at post-cryolesion days 3 and 10. In addition, leucine supplementation minimized the cryolesion-induced decrease in tetanic strength and increase in fatigue in regenerating muscles at post-cryolesion day 10. These beneficial effects of leucine were not accompanied by activation of any elements of the phosphoinositide 3-kinase/Akt/mechanistic target of rapamycin signalling pathway in the regenerating muscles. Our results show that leucine improves myofiber size gain and strength recovery in regenerating soleus muscles through attenuation of protein ubiquitination. In addition, leucine might have therapeutic effects for muscle recovery following injury and in some muscle diseases.

## Introduction

Mammalian skeletal muscle has the ability to adapt to environmental conditions, including a remarkable capacity to regenerate after damage. This regeneration process is highly synchronised and complex, involving the activation of various cellular responses, initially characterised by myofiber necrosis and activation of inflammatory cells [1]. This step is followed by activation of satellite cells, which undergo proliferation, differentiation and fusion to one another or to uninjured portions of the myofiber in order to repair the muscle tissue and restore its function [2], [3], [4].

Recovery of the myofiber cross-sectional area (CSA) is a crucial event in the process of muscle regeneration. Accordingly, studies have shown that the recovery of skeletal muscle after damage is highly regulated by intracellular signalling pathways that control protein turnover, maintaining a balance between the synthesis and degradation of proteins. The activation of elements involved in the mRNA translation process, such as the kinase mechanistic target of rapamycin (mTOR) and its downstream targets, is an essential step for the muscle regenerative response [5], [6].

The phosphoinositide 3-kinase (PI3K)/Akt/mTOR pathway is the major signalling pathway that regulates translation of mRNA in skeletal muscle upon upstream inputs such as growth factors and amino acid availability. The mTOR kinase and its downstream substrates—the 70-kDa ribosomal protein S6 kinase (p70^S6K^); eukaryotic initiation factor (eIF) 4E-binding protein 1 (4E-BP1); and the eIF4F complex, which is composed of eIF4E, eIF4G, and eIF4A—are the crucial elements in the regulation of mRNA translation [7], [8], [9], [10]. During the translation, the small 40S ribosome subunit is recruited to the m^7^G cap structure of the 5′ end of the mRNA, where it scans for the start codon and is assembled into the complete ribosome. Access to the cap structure is provided by eIF4E, which is a major determinant of mRNA efficiency. Hyperphosphorylation of 4E-BP1 releases it from eIF4E and facilitates this binding. In addition, phosphorylation of mTOR activates p70^S6K^, which in turn promotes the activation of secondary structures in the 5′ untranslated region of mRNA to facilitate ribosome scanning [8], [11], [12]. Furthermore, studies have shown that amino acids are able to induce an increase in intracellular Ca^2+^ [Ca^2+^]i; which triggers mTOR and vacuolar protein sorting 34 (Vps34) activation; more specifically the rise in [Ca^2+^]i increases the direct binding of Ca^2+^/calmodulin to a conserved motif in Vps34 that is required for lipid kinase activity and increased mTOR signaling [13], [14].

Molecular mechanisms that modulate protein degradation, such as the ubiquitin-proteasome system (UPS) pathway, the principal agent of proteolytic activity in muscle, also modulate skeletal muscle adaptation over the course of muscle regeneration [15], [16], [17]. During degradation of damaged muscle tissue, ubiquitin conjugates are bound to target proteins via a ubiquitin protein ligase, E3, which presents substrate recognition sites to the ubiquitin molecule [16]. This ubiquitination process is dependent on a forkhead box-O (FOXO) family of transcription factor, such as FOXO3a, which are responsible for transcription activation of E3 ligases [18], [19]. Finally, the termini of ubiquitinated proteins are tagged for degradation in the proteasome [20], [21], [22].

Although skeletal muscle regeneration has been explored carefully at the structural and cellular levels for many years, the identification of strategies to improve this process is on-going. In addition to pharmacological therapies and treatments that have ergogenic effects, certain essential amino acids (especially leucine) have been shown to modulate protein turnover in skeletal muscle, favouring mass gain [23], [24]. Leucine alone is able to induce phosphorylation of mTOR in skeletal muscle, modulating the translation process in a way similar to that achieved by a full complement of essential amino acids. *In vivo* studies have shown that the effects that leucine supplementation has on muscle tissue are associated with the phosphorylation of p70^S6K^ and 4E-BP1, as well as with release of the eIF4F complex [25], [26], [27]. However, the process of muscle protein degradation can be inhibited by leucine supplementation. In fact, it has demonstrated that leucine supplementation, by minimising the expression of E3 ligases [28], attenuates the muscle wasting induced by immobilization. In addition, leucine has no apparent effect on the recovery of protein synthesis after muscle atrophy.

Leucine has anti-catabolic effects, as well as anabolic effects, which increase translational efficiency. Therefore, its use as a therapeutic agent could promote the recovery of skeletal muscle mass after damage. In fact, leucine has been shown to improve the overall morphology of regenerating muscles [29], although the intracellular mechanisms involved have not been fully explored. Therefore, the aim of this study was to gain further insight into the effects that leucine supplementation, started 3 days prior to muscle injury, has on the morphological, molecular, and functional recovery of damaged skeletal muscle in rats. Our results show that leucine improves myofiber size gain and strength recovery in regenerating soleus muscles through attenuation of protein ubiquitination.

## Materials and Methods

This study was carried out in strict accordance with the ethical principles for animal research set forth by the Brazilian College of Animal Experimentation. The protocol was approved by the Ethics in Animal Research Committee of the Institute of Biomedical Sciences at the University of Sao Paulo (Permit Number: 87/2011). Surgical procedures were performed under ketamine and xylazine, or sodium thiopental or tribromoethanol anesthesia, and all efforts were made to minimize suffering. At the end of protocols, all animals were deeply anesthetized and euthanized by cervical dislocation.

### Animals

In this study, we used 2-month-old male Wistar rats (*n* = 48), weighing 283.3±10.8 g. Animals were housed in standard plastic cages in a temperature- and light-controlled environment (24°C; 12/12-h light/dark cycle), with ad libitum access to standard rat chow and water.

### Experimental design

Animals received leucine supplementation or not and were submitted to cryolesion of the soleus muscle of the left hind limb. The contralateral soleus muscle (right hind limb) was used as an intact control [6], [30]. In order to investigate the effects of leucine supplementation on muscle regeneration, muscles were randomly divided into four groups: control (untreated, right hind limb; *n* = 6); leucine supplementation only (Leu, right hind limb; *n* = 6); cryolesion only (Cryo, untreated, left hind limb; n = 6) and leucine supplementation combined with cryolesion (Cryo+Leu, treated, left hind limb; *n* = 6).

Distinct muscles (n = 6; each group) were used in the experiments to measure muscle function. Beginning at 3 days prior to cryolesion and continuing until the end of each experimental period (until post-cryolesion days 1, 3, and 10), leucine (_L_-Leucine; Ajinomoto, Japan) was administered once a day by oral gavage at a dose of 1.35 g/kg body weight [27], [31], [32]. Leucine was dissolved in distilled water and each animal was gavaged with a 5 mL volume of distilled water [27]. In our previous experiments, muscles from saline-gavaged rats did not show morphological changes compared to those from intact animals (data not shown). Prior to cryolesion, the animals were anaesthetized with ketamine and xylazine (95 and 12 mg/kg BW, i.p.), and all efforts were made to minimize suffering.

In each animal, one soleus muscle (left hind limb) was surgically exposed by a lateral incision between the fascias of the anterior and posterior muscle groups until the dorsal surface of the muscle was completely exposed. The cryolesion consisted of one freeze-thaw cycle of the muscle in situ. An iron bar (0.4×0.4 cm) was precooled in liquid nitrogen. The flat side of the bar was brought into full contact with the soleus muscle for 10 s. After the muscle had thawed, the wounds were closed with 6-0 silk sutures. For several min thereafter, the animals were held on a warming plate (37°C) to avoid hypothermia.

On post-cryolesion days 1, 3, and 10, the animals received the last dose of leucine. One hour later [27] each rat was again deeply anesthetized with sodium thiopental (5 mg/100 g BW, i.p.) and the soleus muscles (left and right hind limb) were removed and weighed. Subsequently, animals were euthanized.

### Morphometric and quantitative analyses

After being removed from the animals, the muscles were frozen in melting isopentane and stored in liquid nitrogen. Frozen muscles were cut into 10-µm cross sections on a cryostat (CM3050; Leica, Germany). To reveal the overall morphology, unfixed histological sections were stained with a solution of aqueous toluidine blue and borax (1% w/v for both). The stained sections were analysed under a light microscope (PCM 2000; Nikon, USA). The morphometric and quantitative analyses were conducted with a digitizing unit linked to computer software (Image-Pro Plus; Media Cybernetics, USA). To assess the myofiber cross-sectional area (CSA; µm^2^), a total of approximately 500 myofibers per muscle were measured. In the cryolesion groups, the CSA measurements were obtained only from the regenerating myofiber with centralized nuclei [6]. CSA measurements were expressed as mean ± SD. Myofibers with centralized nuclei were counted in three entire cross sections for each group, and the total area of the section was measured using the software ImagePro Plus. Regenerating myofibers with centralized nuclei were expressed as number per square millimeter (mm^2^). Figures were mounted using Adobe PhotoShop v7.0, with image manipulation being restricted to overall threshold and brightness adjustments.

### Immunostaining

Muscle cross sections to be used for immunodetection of macrophage, collagen type III, and FOXO3a were fixed with 4% paraformaldehyde in 0.2 M phosphate buffer (PB) for 10 min at room temperature, blocked with 0.1 glycine in phosphate-buffered saline (PBS) for 5 min, and permeabilized in 0.2% Triton X-100/PBS for 10 min. The slides were incubated overnight in a moisture chamber at 4°C with a solution containing the primary antibodies together with 3% normal goat serum and 0.3% Triton X-100/0.1 M PB. After the slides had been washed (three 10-min washes with 0.1 M PB), a solution containing the respective secondary antibodies and 0.3% Triton X-100/0.1 M PB was added, and the slides were maintained in this solution for 2 h in a dark room. The slides were again washed in 0.1 M PB (three 10-min washes), after which they were mounted with Vectashield mounting medium containing 4′,6-diamidino-2-phenylindole (Vector Laboratories) and coverslipped.

The primary antibodies used were: mouse monoclonal anti-macrophage MAC387 (1∶200; Abcam Inc, Cambridge, MA, USA); mouse monoclonal anti-Collagen III (1∶250; Abcam Inc, Cambridge, MA, USA); and, rabbit polyclonal anti-FOXO3a (1∶300; Abcam Inc, Cambridge, MA, USA). The following secondary antibodies were: FITC-conjugated goat anti-mouse (1∶200; Jackson ImmunoResearch, West Grove, PA, USA); CY3 donkey anti-mouse (1∶200; Jackson ImmunoResearch, West Grove, PA, USA); and, CY2 goat anti-rabbit (1∶200; Jackson ImmunoResearch, West Grove, PA, USA).

Macrophages and the nuclei positive for FOXO3a (activated FOXO3a) were counted in three entire cross sections for each group, and the total area of the section was measured using the software ImagePro Plus. The volume of muscle samples was calculated as the product of the CSA of the section and the section thickness (10 µm). The quantity of macrophages and FOXO3a positive nuclei was expressed as number per cubic millimeter [33].

A planimetry system was used for the analysis of the intramuscular collagen type III density by scoring the points containing 500 line intersections per field. The coincident points in the endomysium and perimysium in three areas per section in four sections per animal corresponded to a total of 6,000 points per animal. The relative area of collagen III in muscle tissue (area density) was calculated by dividing the sum of the number of the coincident points in the straight line intersections in the connective tissue by the total number of points and was expressed as a percentage of whole muscle cross sections [34].

The stained sections were analysed in a Nikon microscope (PCM2000, Nikon, Melville, New York, USA). Figures were mounted using Adobe PhotoShop v7.0, with image manipulation being restricted to overall threshold and brightness adjustments.

### Western blot analysis

To quantify the expression of elements of the PI3K/Akt/mTOR pathway, soleus muscles were homogenised in an extraction solubilisation buffer, composed of 90 mM KCl, 10 mM 4-2-hydroxyethyl-1-piperazineethanesulfonic acid, 3 mM MgCl^2+^, 5 mM ethylenediaminetetraacetic acid (EDTA), 1% glycerol, 1 mM dithiothreitol, 0.04% sodium dodecyl sulfate, proteinase, and phosphatase inhibitor cocktail (1∶100; Sigma-Aldrich, USA). For studies to detect the amount of ubiquitinated proteins, soleus muscles were homogenized in an extraction solubilisation buffer, composed of 0.625% non-ionic detergent (Nonidet P-40; Sigma-Aldrich, USA), 0.625% sodium deoxycholate, 6.25 mM sodium phosphate, 1 mM EDTA (pH 7.4) containing 10 µg/ml of protease inhibitor cocktail (Sigma-Aldrich, USA). Homogenates were centrifuged at 12,000×*g* for 10 min at 4°C, the supernatant was collected, and protein was quantified by Bradford assay (Bio-Rad, USA) with bovine serum albumin as a standard [35]. Equal amounts of protein (50 µg) were separated on 6–15% sodium dodecyl sulfate-polyacrylamide gels, electrophoresed, and transferred to a nitrocellulose membrane (Bio-Rad, USA). The membranes were stained with Ponceau S to determine the protein content and then rinsed with Tris-buffered saline/Tween solution (0.5 M NaCl; 50 mM Tris-HCl, pH 7.4; and 0.1% Tween 20). Membranes were incubated overnight at 4°C with primary antibodies. After a 30 min wash in Tris-buffered saline/Tween solution, membranes were incubated with secondary antibodies for 1 h at room temperature. The membranes were again washed for 30 min in Tris-buffered saline/Tween solution. Detection of the labelled proteins was achieved using the enhanced chemiluminescence system (ECL; Amersham, UK) and autoradiography. Densitometry analysis was performed by using ImageJ software (Scion Corp., National Institutes of Health, Bethesda, Maryland, USA). Experiments were performed on four separate samples from each group.

The primary antibodies used for Western blotting were rabbit polyclonal antibodies raised against mTOR, phospho-mTOR at Ser^2448^ residue, phospho-p70^S6K^ at Ser^371^ and Thr^389^ residue, 4E-BP1, phospho-4E-BP1 at Thr^70^ and Thr^37/46^ residues, and eIF4E (1∶1,000; Cell Signaling Technology, USA). Targeted bands were normalised to glyceraldehyde-3-phosphate dehydrogenase (1∶1,000; Cell Signaling Technology, USA). In addition, we used a rabbit polyclonal antibody raised against ubiquitin (1∶1,500; Boston Biochem, USA). The secondary antibody used for all Western blots was peroxidase-conjugated goat anti-rabbit IgG (AffiniPure, 1∶10,000; Jackson ImmunoResearch Laboratories Inc., USA).

### In vivo muscle function experiments

On post-cryolesion day 10, animals were anaesthetized with tribromoethanol (20 mg/100 g body weight, i.p.). The sciatic nerve was then exposed through a lateral incision on the thigh, and an electrode was connected. The innervations of the sciatic nerve to the soleus muscle were carefully isolated from those originating from other nerves. The distal lateral and medial tendons of the gastrocnemius muscle were surgically separated from the soleus tendon to avoid injuries to the innervations of soleus muscles in the mid-belly region between the soleus and gastrocnemius muscles. The rats were then placed on an acrylic platform with a metallic bar crossing the knee in order to immobilize the limb. The soleus tendon was connected to a force transducer coupled to a computer that was used in order to collect and analyse data related to the strength generated by the muscle contraction. Muscle twitch strength and tetanic force were recorded using a data acquisition system (Biopac Systems, USA). Muscle strength was analysed using the AcqKnowledge program, version 3.9.1.6 (Biopac Systems, USA). Rats were submitted to external warming in order to maintain core temperature throughout the procedure.

At the start of the experiment, the muscle was set to the optimum length (*L_0_*, defined as the length resulting in maximum twitch strength). There was a 2-min rest period between stimuli [36]. To achieve the maximal plateau strength with the minimal frequency, we chose to use stimuli of 350 Hz for measuring the maximum isometric tetanic strength and 200 Hz for measuring fatigue [37].

Based on Chan & Head [37], isolated twitches (0.2 Hz) were generated over a 2-min period, followed by a pre-fatigue maximum tetanic contraction (induced at 350 Hz for 2 s) in each soleus muscle. We then performed a fatigue protocol, which consisted of ten 2-s stimulations (at 200 Hz tetanus), each followed by a 4-s rest. At the end of the fatigue protocol, a 2-min rest-period was given to the muscle by stimulating it at 0.2 Hz, followed by a post-fatigue maximum tetanic contraction (induced at 350 Hz for 2 s). We observed no differences among the groups in terms of twitch parameters, such as the time-to-peak and half-relaxation time (data not shown). Tetanic strength is expressed in millinewtons. Development of muscle fatigue was measured at four time points (1^st^, 4^th^, 7^th^, and 10^th^ contractions).

### Statistical analysis

Data are presented as mean and standard deviation. We used mixed models for repeated measures in order to evaluate the effects that cryolesion and leucine supplementation have on maximum tetanic strength and the development of muscle fatigue. Analysis of variance (the general linear model) was used in order to evaluate the effects that cryolesion and leucine supplementation have on body weight, muscle weight, CSA, area density of collagen, protein expression analysis, and quantification of inflammatory cells, and FOXO3a positive nuclei. Student *t*-test was used in order to evaluate the effects that leucine supplementation has on incidence of myofibers with centralized nuclei. Whenever a significant *F*-value was obtained, Tukey's *post-hoc* test was performed for multiple comparison purposes (SAS 9.2 software; SAS Institute Inc., USA). Values of *p*<0.05 were considered statistically significant.

## Results

### Body weight, muscle weight, and muscle histology

Body weight remained unchanged in all of the animals evaluated. The food consumption was also unaltered in all groups (data not shown). In the control groups, soleus muscle weight was unaltered over the course of 3 and 10 days of observations (Table 1). However, there was a rise in soleus muscle weight from Leu group at day 10 when compared to that from day 1 (33%, *p*<0.05, Table 1). Damaged soleus muscles in the Cryo and Cryo+Leu groups showed a significant increase in their weights when compared to those from Control group on post-cryolesion day 1 (30% increase in both groups, *p*<0.05, Table 1). On post-cryolesion day 3, soleus muscle weights from Cryo and Cryo+Leu groups were decreased when compared to those from day 1 (31% in both, Table 1). On post-cryolesion day 10, soleus muscle weight was reduced in the Cryo group (31%), but not in Cryo+Leu group when compared to that from Cryo and Cryo+Leu groups, respectively, on day 1 (Table 1).

**Table 1:** Soleus muscle weight and body weight of rats at post-cryolesion days 1, 3, and 10.

Post-cryolesion time point	Muscle weight (g)				Body weight (g)	
	Mean±SD				Mean±SD	
	Group				Group	
	Control	Leu	Cryo	Cryo+Leu	Control/Cryo	Leu/Cryo+Leu
Day 1	0.10±0.01	0.09±0.03	0.13±0.02^a^	0.13±0.02^a^	293.2±17.8	276.8±8.7
Day 3	0.10±0.01	0.09±0.06	0.09±0.02^b^	0.09±0.01^b^	282.1±23.8	266.4±11.7
Day 10	0.11±0.03	0.12±0.06^b^	0.09±0.01^b^	0.11±0.02	292.3±6.0	291.9±3.7

Control: intact muscles; Leu: muscles supplemented with leucine; Cryo: cryolesioned muscles; Cryo+Leu: cryolesioned muscles supplemented with leucine;

Control/Cryo: animals that had the left soleus muscle cryolesioned and the right soleus muscles used as control; Leu/Cryo+Leu: animals that were supplemented with leucine and had the left soleus muscle cryolesioned and the right soleus muscles used as control. ^a^ *p*<0.05 vs. Control and Leu in the same post-cryolesion time point; ^b^ *p*<0.05 vs. matched group on Day 1 after cryolesion (analysis of variance followed by Tukey's post hoc test for multiple comparison.

**Figure 1:**
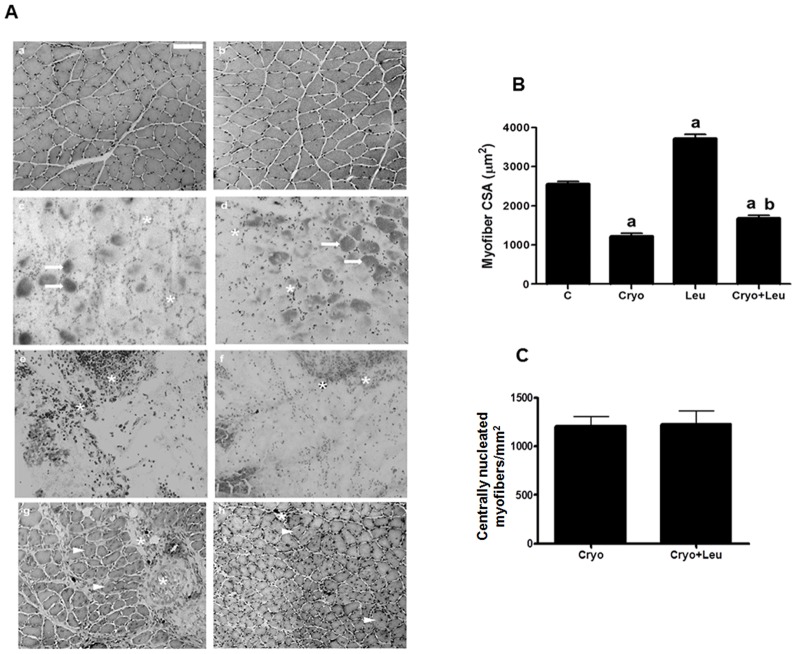
Effects of leucine supplementation on skeletal muscle morphology. **A**: Histological features of soleus muscle cross sections. *a*: C, intact control muscle; *b*: Leu, control muscle supplemented only with leucine from the time point of post-cryolesion day 10; *c*: Cryo, damaged muscle analysed on post-cryolesion day 1; *d*: Cryo+Leu, leucine- supplemented damaged muscle analysed on post-cryolesion day 1; *e*: Cryo, damaged muscle analysed on post-cryolesion day 3; *f*: Cryo+Leu, leucine-supplemented damaged muscle analysed on post-cryolesion day 3; *g*: Cryo, damaged muscle analysed on post-cryolesion day 10; and *h*: Cryo+Leu, leucine-supplemented damaged muscle analysed on post-cryolesion day 10. Note that the intact control muscle (*a*) and the leucine supplemented-only muscle (*b*) have normal morphology. At post-cryolesion day 1, note the hyper-contracted fibers, i.e., fibers with dark regions (arrows in *c* and *d* of panel A), which result from myofiber disruption. In addition, there are clear spaces between the cells, indicating regions of totally destroyed myofibers and presence of inflammatory cells (asterisks). At post-cryolesion day 3, note the intense inflammatory process in Cryo and Cryo+Leu group muscles (asterisks in *e* and *f* of panel A). At post-cryolesion day 10, Cryo and Cryo+Leu group muscles have regenerating myofibers with centralized nuclei (arrowheads in *g* and *h* of panel A) and apparently less inflammatory infiltration in the Cryo+Leu group (asterisks in *g* and *h*). Toluidine blue staining. Bar = 50 µm. **B**: Myofiber cross-sectional area (CSA; µm^2^) of soleus muscles. *C*: intact control muscles; *Leu*: control muscles supplemented only with leucine from the time point of post-cryolesion day 10; *Cryo*: damaged muscles analysed on post-cryolesion day 10; and, *Cryo+Leu*: leucine-supplemented damaged muscles analysed on post-cryolesion day 10. Data are presented as mean±SD (n = 6). ^a^ *p*<0.05 vs. C and Leu; ^b^ *p*<0.05 vs. Cryo. **C**: Incidence of myofibers with centralized nuclei per area (mm^2^) of soleus muscle tissue. *Cryo*: damaged muscles analysed on post-cryolesion day 10; *Cryo+Leu*: leucine-supplemented damaged muscles analysed on post-cryolesion day 10. Data are presented as mean±SD (n = 6).

Histological cross sections of soleus muscles were stained with toluidine blue and microscopically analysed on the days corresponding to post-cryolesion days 1, 3, and 10 (Figure 1A). The intact control muscle exhibited polygonal myofibers with peripheral nuclei, i.e., a normal tissue structure (Figure 1A-*a*). On leucine supplementation day 10, the myofiber CSA was 45% larger in a soleus muscle from a Leu group than in one obtained from a control group rat (*p*<0.05, Figure 1A-*b*, and B).

On post-cryolesion day 1, the muscles from the Cryo and Cryo+Leu groups showed significant signs of damage, as evidenced by the presence of hyper-contracted myofibers, empty spaces between myofibers, and inflammatory cells; which indicated myonecrosis, tissue disruption, and oedema (Figure 1A-*c*, and A-*d*). These markers of damage reflected on increased muscle weight in both Cryo and Cryo+Leu groups (30% in both, Table 1), as previously mentioned. On post-cryolesion day 3, the muscles from the Cryo and Cryo+Leu groups still presented extensive damage, indicated by clear areas among the myofiber and extensive inflammatory cell infiltration (Figure 1A-*e*, and A-*f*). However the oedema significantly reduced, as shown by unaltered muscle weights in both Cryo and Cryo+Leu groups when compared to those from control group (Table 1).

Soleus muscles evaluated on post-cryolesion day 10 exhibited an apparent reduction in the inflammatory process when compared with those evaluated on post-cryolesion days 1 and 3. In addition, the Cryo and Cryo+Leu group soleus muscles showed regenerating myofibers with centralised nuclei, a feature not observed in the corresponding control muscles (Figure 1A-*g* and A-*h*). Cryo soleus muscles showed a significantly (53%) smaller CSA when compared with the corresponding intact control muscles (*p*<0.05, Figure 1A-*g* and B). At the same time point, the Cryo+Leu group soleus muscles showed inflammatory infiltration affecting a smaller area (Figure 1A-*h*) and the myofibers of those muscles were 40% larger, when compared with Cryo group muscles (*p*<0.05, Figure 1B). In addition, soleus muscle from Cryo and Cryo+Leu groups analysed on post-cryolesion day 10 showed a similar number of regenerating myofibers with centralized nuclei (Figure 1C).

### Area density of collagen type III and incidence of macrophages are reduced in regenerating muscles supplemented with leucine

**Figure 2:**
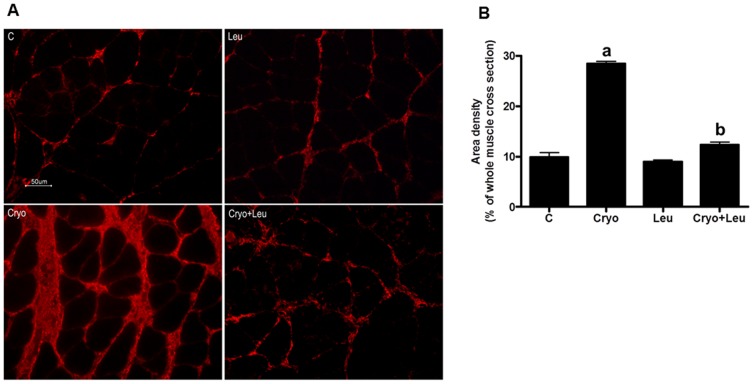
Leucine supplementation reduces area density of collagen type III in regenerating muscles. **A**: Soleus muscle cross sections immunostained for collagen type III. *C*: intact control muscle; *Leu*: control muscles supplemented only with leucine from the time point of post-cryolesion day 10; *Cryo*: damaged muscle analysed on post-cryolesion day 10; and, *Cryo+Leu*: leucine-supplemented damaged muscle analysed on post-cryolesion day 10. Bar = 50 µm. **B**: Area density of collagen type III (percentage of the whole muscle cross section) in soleus muscles. Data are presented as mean±SD (n = 5). ^a^ *p*<0.05 vs. C and Leu; ^b^ *p*<0.05 vs. Cryo.

Histological cross sections of soleus muscles collected on post-cryolesion day 10, were immunostained against collagen type III and analysed. Cryo group showed an increased area density of collagen type III when compared to that from C and Leu groups (180% of increase, *p*<0.05, Figure 2A and B). There was a decrease of area density of collagen type III in Cryo+Leu group when compared to that from Cryo group (60%, *p*<0.05, Figure 2A and B).

**Figure 3:**
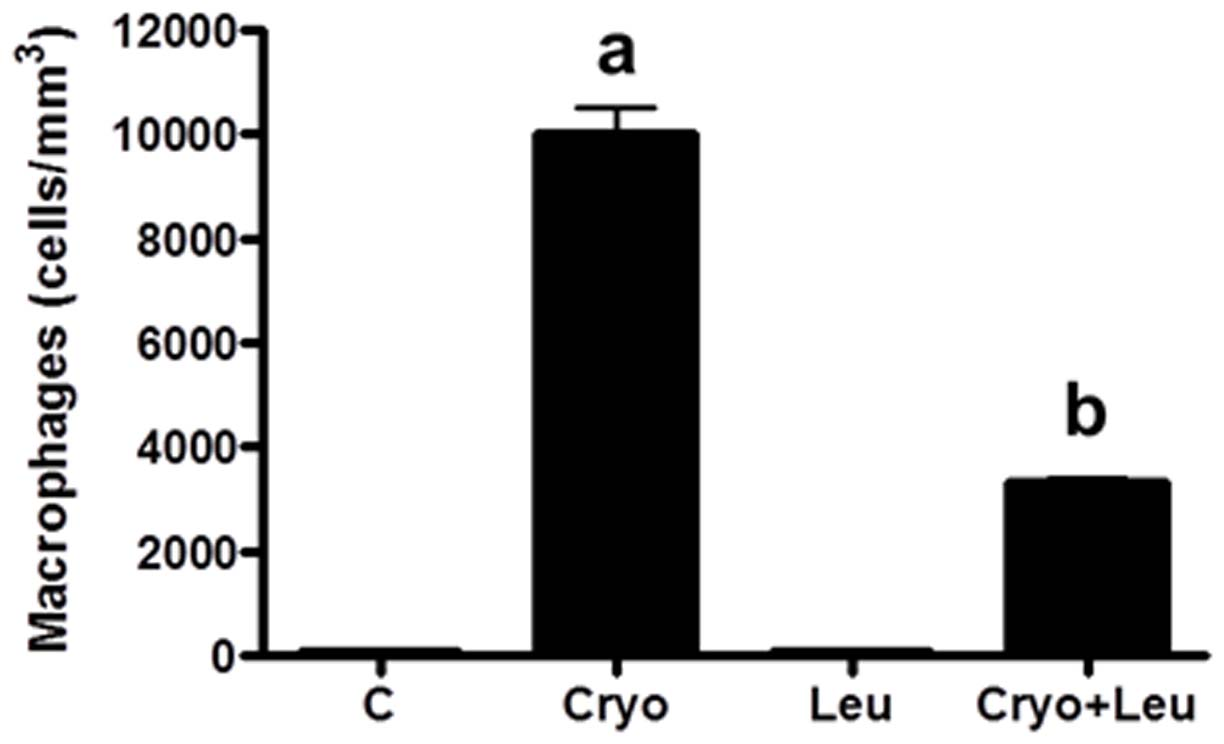
Leucine supplementation reduces incidence of macrophages. Number of macrophages per cubic millimeter of soleus muscles. *C*: intact control muscles; *Leu*: control muscles supplemented only with leucine from the time point of post-cryolesion day 3; *Cryo*: damaged muscles analysed on post-cryolesion day 3; and, *Cryo+Leu*: leucine-supplemented damaged muscles analysed on post-cryolesion day 3. Data are presented as mean±SD (n = 5). ^a^ *p*<0.05 vs. C and Leu; ^b^ *p*<0.05 vs. Cryo.

In addition, the number of macrophages in soleus muscles from Cryo+Leu group evaluated on post-cryolesion day 3 was decreased when compared to that from Cryo group (60%, *p*<0.05, Figure 3).

### Expression of elements within the PI3K/Akt/mTOR signalling pathway

Since we found no effect of leucine in the expression of elements within the PI3K/Akt/mTOR pathway, i.e. phosphorylated p70^S6K^ at Ser^371^ and Thr^389^ residues, in regenerating muscles from 3 days post-cryolesion (data not shown), and muscles from 1 and 3 days post-cryolesion had similar morphological features of early damage; we decided to focus our western blot analyses for elements within the PI3K/Akt/mTOR pathway in muscles from 10 days post-cryolesion.

**Figure 4:**
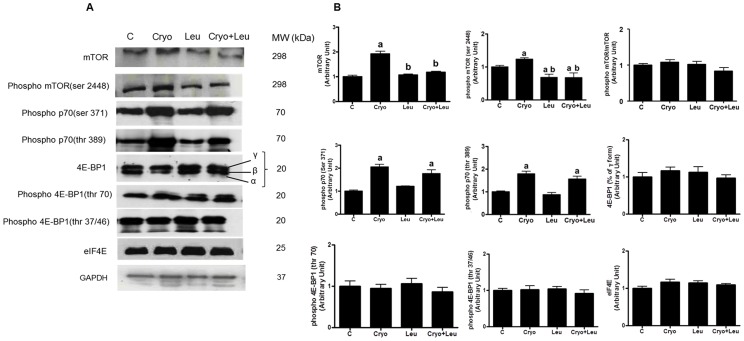
Leucine supplementation does not activate PI3K/Akt/mTOR signalling pathway in regenerating muscles. **A**: Representative Western blots of elements from the PI3K/Akt/mTOR signalling pathway. Muscle groups are identified at the top. *C*, intact control muscle; *Leu*, control muscles supplemented only with leucine from the time point of post-cryolesion day 10; *Cryo*, damaged muscle analysed on post-cryolesion day 10; and *Cryo+Leu*, leucine-supplemented damaged muscle analysed on post-cryolesion day 10. **B**: Densitometry analyses of elements from the PI3K/Akt/mTOR signalling pathway. Blots were reacted with antibodies specific for mTOR; phosphorylated mTOR at Ser^2448^ residue; phosphorylated p70 at Ser^371^ and Thr^389^ residues; 4E-BP1; p-4E-BP1 at Thr^70^ and Thr^37/46^ residues; eIF4E; and glyceraldehyde-3-phosphate dehydrogenase (GAPDH), which was used as standard. Mw: molecular weight. Data are presented as mean±SD (n = 4). ^a^ *p*<0.05 vs. C and Leu; ^b^ *p*<0.05 vs. Cryo.

We found it surprising that the leucine supplemented-only group had a slight decrease on mTOR phosphorylation at Ser^2448^ residue (26%, *p*<0.05, Figure 4A and B) and no changes in the expression of other PI3K/Akt/mTOR pathway elements analysed, including mTOR, p-p70^S6K^ at Ser^371^ and Thr^389^ residues; 4E-BP1; p-4E-BP1 at Thr^70^ and Thr^37/46^ residues; and eIF4E (Figure 4A and B). On post-cryolesion day 10, muscles from Cryo group showed an increase in the expression of mTOR, p-mTOR (at Ser^2448^), and p-p70 (at Ser^371^ and Thr^389^) when compared with that observed for the intact control muscle (90%, 30%, 100% and 80%; respectively, *p*<0.05, Figure 4A and B). In addition, the expression of mTOR and p-mTOR (at Ser^2448^) was significantly decreased in the Cryo+Leu group muscles (42% and 45%, *p*<0.05, Figure 4A and B); and the cryolesion-induced increase in p-p70 (at Ser^371^ and Thr^389^) was unaffected in the Cryo+Leu group muscles (Figure 4A and B).

### Attenuation of ubiquitinated protein accumulation and of FOXO3a activation on regenerating muscles supplemented with leucine

**Figure 5:**
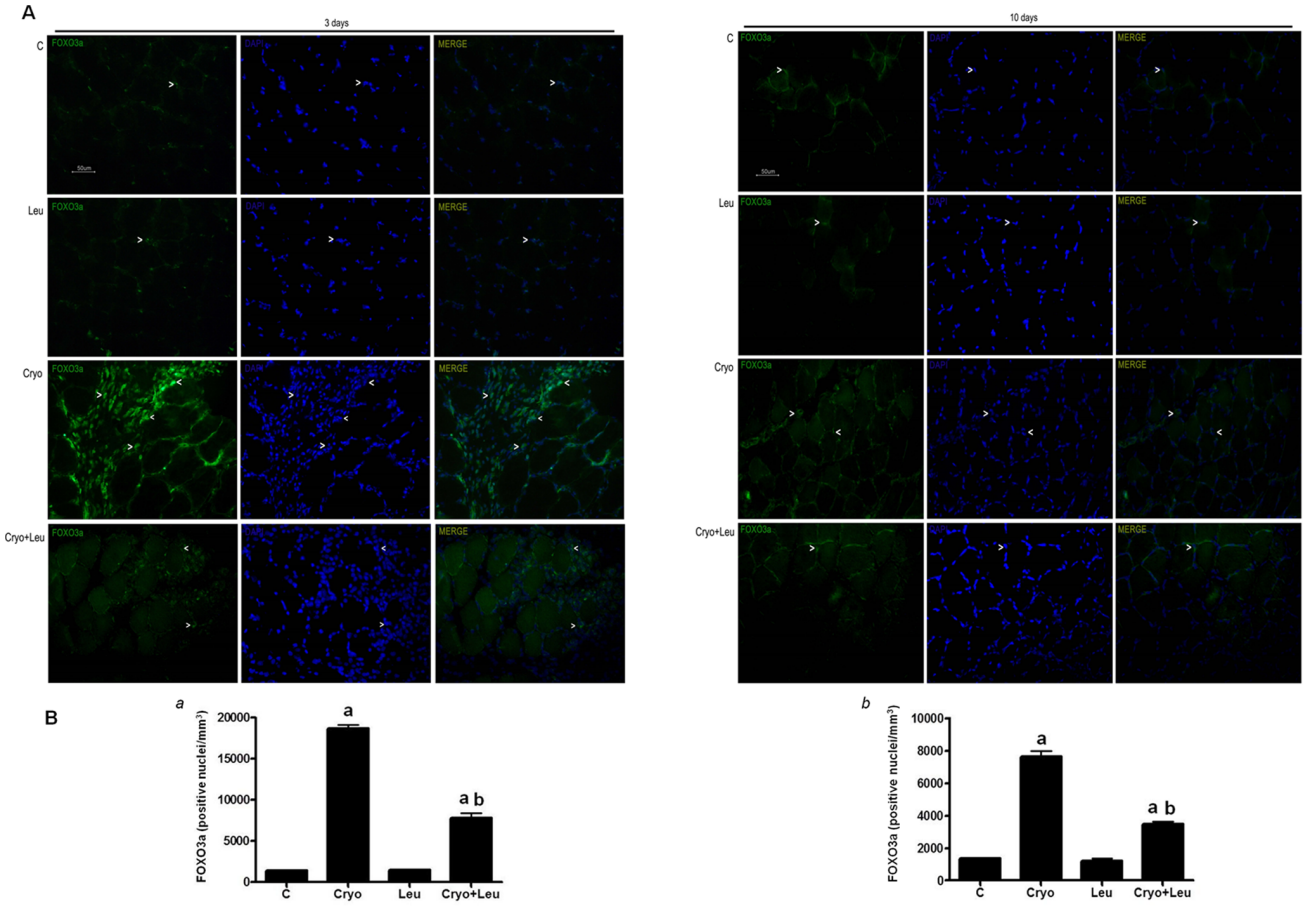
Leucine supplementation reduces FOXO3a activation in regenerating muscles. **A**: Soleus muscle cross sections immunostained for FOXO3a. Activation of FOXO3a was assessed by quantifying FOXO3a positive nuclei per cubic millimeter. Post-cryolesion times are identified at the top. *C*: intact control muscle; *Leu*: control muscles supplemented only with leucine from the time points of post-cryolesion day 3 and 10, respectively; *Cryo*: damaged muscle analysed on post-cryolesion days 3 or 10, respectively; and, *Cryo+Leu*: leucine-supplemented damaged muscle analysed on post-cryolesion days 3 or 10, respectively. Arrowheads indicate FOXO3a, DAPI (nucleus staining), and merge of FOXO3a and DAPI. Bar = 50 µm. **B**: Number of FOXO3a positive nuclei per cubic millimeter of soleus muscles from post-cryolesion day 3 (panel *a*) and 10 days (panel *b*). Data are presented as mean±SD (n = 5). ^a^ *p*<0.05 vs. C; ^b^ *p*<0.05 vs. Cryo group.

Activation of FOXO3a transcription factor was assessed by quantifying the number of nuclei positive for FOXO3a in skeletal muscle tissues collected on post-cryolesion days 3 and 10. On post-cryolesion day 3, muscles from Cryo group showed a robust increase in the number of nuclei positive for FOXO3a, when compared to those from control group (13 folds, *p*<0.05, Figure 5A and B). Muscles from Cryo+Leu group had a decreased activation of FOXO3a when compared to those from Cryo group (58%, *p*<0.05, Figure 5A and B).

On post-cryolesion day 10, muscles from Cryo group still showed a great increase in activation of FOXO3a, when compared to those from control group (5.6 folds, *p*<0.05, Figure 5A and B). Similarly to the results from 3 days post-cryolesion, there was a reduced activation of FOXO3a in muscles from Cryo+Leu group when compared to those from Cryo group (55%, *p*<0.05, Figure 5A and B).

**Figure 6:**
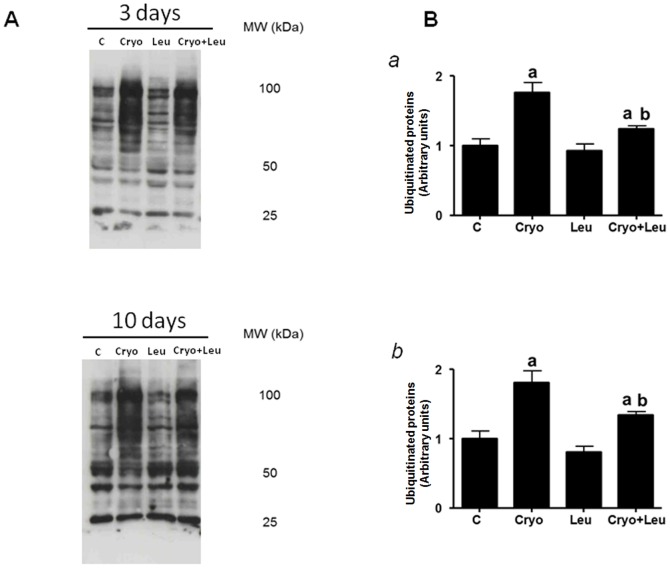
Leucine supplementation reduces ubiquitinated protein accumulation. **A**: Representative Western blots of ubiquitinated proteins. Samples were analysed at two different time points of experimental protocol (3 and 10 days post-cryolesion). Muscle groups and experimental time points are identified at the top. *C*, intact control muscle; *Leu*, control muscles supplemented only with leucine from the time points of post-cryolesion day 3 and 10, respectively; *Cryo*, damaged muscle analysed on post-cryolesion days 3 and 10, respectively; and *Cryo+Leu*, leucine-supplemented damaged muscle analysed on post-cryolesion days 3 and 10, respectively. **B**: Densitometry analyses of ubiquitinated proteins at 3 (panel *a*) and 10 days (panel *b*) of experimental protocol. Blots were reacted with antibody specific for ubiquitin. Mw: molecular weight. Data are presented as mean±SD (n = 4). ^a^ *p*<0.05 vs. C and Leu; ^b^ *p*<0.05 vs. Cryo.

The amount of ubiquitin-conjugated proteins in the soleus muscle was notably elevated in the Cryo group than in the control group on post-cryolesion days 3 and 10 (70% and 65%; respectively, *p*<0.05, Figure 6A and B). There was no difference between the muscles from the Leu group and the intact control muscles. However, at the same time points (post-cryolesion days 3 and 10), the cryolesion-induced increase in the amount of ubiquitinated protein was attenuated in the Cryo+Leu group muscles (30% and 25%; respectively, *p*<0.05, Figure 6A and B).

### Leucine supplementation prevents loss of function in regenerating muscles

**Figure 7:**
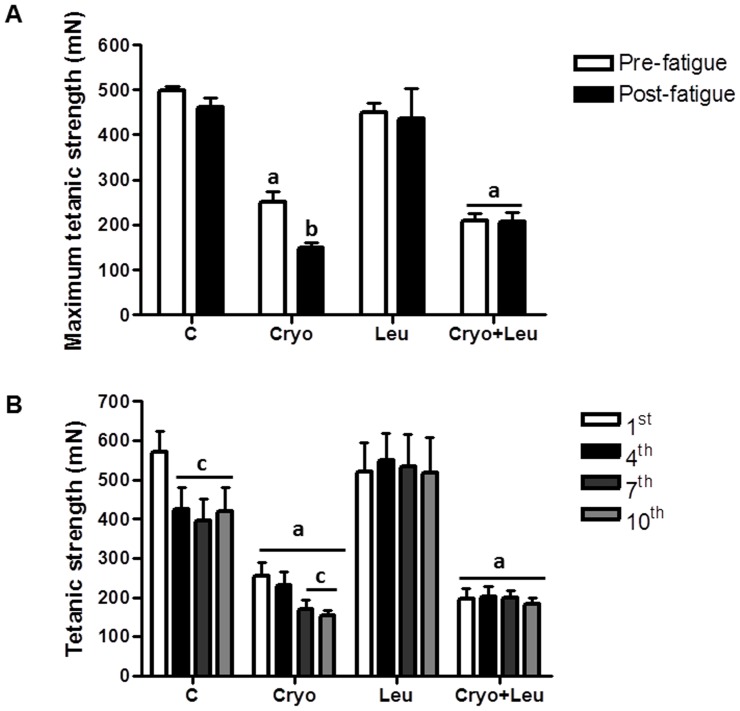
Leucine supplementation minimizes loss of function in regenerating muscles. **A**: Maximum tetanic strength at pre-fatigue and post-fatigue protocol in animals analysed on post-cryolesion day 10. **B**: Development of muscle fatigue measured at four time points (1^st^, 4^th^, 7^th^, and 10^th^ contractions) in animals analysed at 10 days of experimental protocol. *C*: intact control muscles; *Leu*: control muscles supplemented only with leucine from the time point of post-cryolesion day 10; *Cryo*: damaged muscles analysed on post-cryolesion day 10; and, *Cryo+Leu*: leucine-supplemented damaged muscles analysed on post-cryolesion day 10. Muscle strength is expressed in millinewtons (mN). Data are presented as mean±SD (n = 6). ^a^ *p*<0.05 vs. C and Leu; ^b^ *p*<0.05 vs. Cryo pre-fatigue tetanic stimulus; ^c^ *p*<0.05 vs. 1^st^ contraction in fatigue protocol.

The influence of leucine supplementation on the functional recovery of regenerating soleus muscles was evaluated through the analysis of the maximum tetanic strength and the development of muscle fatigue on post-cryolesion day 10. There was no difference between the control and Leu groups in terms of the results of pre and post-fatigue tetanic strength stimulations. However, comparing Cryo group muscles and control group muscles, strength production at pre-fatigue tetanic stimulus was 50% lower in the former (*p*<0.05, Figure 7A). In addition, the Cryo group muscles showed a 42% decrease in strength production from pre-fatigue to post-fatigue tetanic stimulus (*p*<0.05, Figure 7A). Although strength production at pre-fatigue tetanic stimulus was also lower in the Cryo+Leu group muscles than in the control group muscles (58% lower; *p*<0.05, Figure 7A), the decrease in strength production from pre-fatigue to post-fatigue tetanic stimulus seen in the Cryo group muscles was not observed in the Cryo+Leu group muscles (Figure 7A).

At four time points during the fatigue protocol (1^st^, 4^th^, 7^th^, and 10^th^ contractions), the strength of the soleus muscle was measured. The control muscle significantly decreased muscle strength beginning at the 4^th^ contraction stimulus of the fatigue protocol (a 26% decrease vs. the 1^st^ contraction), and persisting at the 7^th^ and 10^th^ contractions (31% and 27%, respectively; *p*<0.05, Figure 7B). However, in the Leu group, soleus muscle strength did not vary at the 1^st^, 4^th^, 7^th^, and 10^th^ contractions of fatigue protocol (Figure 7B). Muscle strength at the 1^st^, 4^th^, 7^th^, and 10^th^ contractions were significantly (56%, 46%, 57%, and 63%) lower in the Cryo group than in the respective contractions in control group (*p*<0.05, Figure 7B). In the Cryo group, muscle strength was also significantly (∼37%) lower at the 7^th^ and 10^th^ contractions in comparison with the 1^st^ contraction (*p*<0.05, Figure 7B). In addition, muscle strength at the 1^st^, 4^th^, 7^th^, and 10^th^ contractions were lower in the Cryo+Leu group (62%, 52%, 50%, and 57%) than in the respective contractions in control group (*p*<0.05, Figure 7B). However, as in our analysis of strength production, the Cryo+Leu group did not present a decrease in muscle strength at the 4^th^, 7^th^ and 10^th^ contractions, in comparison with that observed at the 1^st^ contraction (Figure 7B).

## Discussion

Skeletal muscle regeneration is an essential process that contributes to the maintenance of muscle mass and function throughout life. Given that muscle mass regulation is key to the success of the muscle regeneration process, supplementation with leucine, an ergogenic agent, represents a good strategy for maximizing muscle mass recovery after various events related to muscle disorders. In the present study, we have shown that leucine supplementation, started 3 days prior to cryolesion, does not protect the muscle against injury, however can benefit the structure and function of regenerating muscle through attenuation of protein ubiquitination.

Initially, we observed that although leucine supplementation increased myofiber size in uninjured soleus muscle, it did not alter muscle weight when compared to control. Previous studies corroborate our data, because long-term leucine supplementation (10 days to 8 weeks of administration, at the same dose utilized in the present study) does not affect the weight of the gastrocnemius or soleus muscles [38], [39], [40]. This effect might be related to a leucine-mediated decrease in the expression of proteins that constitute the extracellular matrix.

A recent study has shown that the internal region of the proteoglycan decorin, which is rich in leucine, interacts with low-density lipoprotein receptor-related protein-1, modulating transforming growth factor beta (TGF-β)-dependent signalling, and consequently inhibiting the TGF-β-dependent fibrotic response in skeletal muscles [41]. In order to address this issue, we evaluated the expression of collagen type III, an extracellular matrix protein present mainly on endomysium and perimysium of skeletal muscle tissue [42], [43]. Although leucine was unable to attenuate the expression of collagen type III in uninjured muscles, other proteins from extracellular matrix might be more responsive to leucine. Accordingly, uninjured muscles subjected to the longest leucine treatment (Day 10, Table 1) increased their weight compared to those that received the shortest one (Day 1, Table 1); therefore even though uninjured muscles are less responsive to leucine, the leucine effects seem to reflect on muscle weight over the course of approximately 10 days. On the other hand, under the regenerative process, leucine significantly reduced the intramuscular expression of collagen type III, which may contribute to a more effective repair of the satellite cell niche and its vascular and neural networks.

In line with the beneficial effect of leucine on reconstitution of extracellular matrix component, leucine was able to robustly reduce macrophage infiltration at 3 days post-cryolesion. According to Nicastro et al. [44], branched-chain amino acids, which include leucine, may influence the inflammatory status of a tissue through transamination of glutamate in order to increase synthesis of glutamine, an amino acid highly consumed by inflammatory cells under pathological conditions. Furthermore, leucine functions as a nitrogen donor for the production of glutamine in skeletal muscle [45]; therefore the regenerating muscles supplemented with leucine might have an acceleration of inflammatory process installation and a more efficient function of immune cells stimulated by increased amino acid substrates. Future studies should deepen the investigation regarding molecular mechanisms mediated by leucine in inflammatory process.

We also found that leucine supplementation resulted in an increase in the size of regenerating myofibers on post-cryolesion day 10. We investigated this effect, in order to determine whether it was related to the activation of elements involved in the PI3K/Akt/mTOR protein synthesis pathway. As expected, we found that, at 10 days after cryolesion, there was activation of mTOR and p70^S6K^ in regenerating muscles. As demonstrated by [6], [30], post-cryolesion day 10 is a time point characterized by intense protein synthesis, together with activation of mTOR and p70^S6K^, in order to recover muscle mass. However, we observed no 10-day post-cryolesion changes in 4E-BP1 phosphorylation or eIF4E, suggesting that the protein synthesis does not involve the activation of translation initiation factors at this time point in the muscle regeneration process.

In uninjured muscles, leucine supplementation did not induce activation of any of the PI3K/Akt/mTOR pathway elements evaluated. Various studies have shown that a single bolus of leucine is able to stimulate the PI3K/Akt/mTOR pathway [27], [31], [32], [46]. However, studies of long-term leucine supplementation (5–8 weeks of treatment) have demonstrated that the activation of this pathway in the soleus muscle is not sustained over time [38], [39], a finding in agreement with those of the present study. In fact, Suryawan et al. showed that leucine-induced stimulation of muscle protein synthesis is not maintained for prolonged periods; because there is a decay in the circulating essential amino acids, which are utilised for protein synthesis [47]. Those authors found that although the activation of translation initiation factors was preserved at 2-h after an infusion of leucine, the stimulation of muscle protein synthesis was not, because of the leucine-induced reduction in the concentrations of essential amino acids [47]. In the present study, we show for the first time that leucine supplementation slightly decreased p-mTOR (at Ser^2448^) expression, and had no effect on other elements of PI3K/Akt/mTOR pathway in regenerating muscles at post-cryolesion day 10. Accordingly, Baptista et al. [28] observed that leucine supplementation did not alter protein synthesis in soleus muscles after 7 days of immobilization. These results lead us to the interpretation that the role of leucine in the recovery of regenerating muscle mass is independent of protein synthesis modulation and dependent on muscle proteolysis activation.

In order to test the hypothesis that leucine reduces protein degradation, thus favouring muscle mass gain, we determined FOXO3a activation and the amount of ubiquitinated proteins. As previously demonstrated, protein ubiquitination increases at 1–14 days after muscle damage [6], which is in agreement with our results showing that regenerating muscles had increased FOXO3a activation, which may lead to transcription activation of E3 ligases [18], [19], and subsequent elevated amount of ubiquitinated proteins on post-cryolesion days 3 and 10.

We found that leucine supplementation diminished FOXO3a activation and the amount of ubiquitinated proteins in the soleus muscle at post-cryolesion days 3 and 10, suggesting that muscle proteolysis is attenuated by leucine. This finding is in agreement with those of Baptista et al. [28], who showed that leucine minimizes E3 ligase gene expression and attenuates overall protein ubiquitination in soleus muscles after 7 days of immobilization. In addition, a single leucine infusion has been shown to decrease chymotrypsin and trypsin-like activities, as well as proteasome-dependent proteolysis, in the skeletal muscles of 8-month-old rats [40].

In order to determine whether the morphological alteration induced by leucine had a functional impact, we analysed muscle contraction at post-cryolesion day 10. As has previously been demonstrated [6], [30], we found that cryolesion provoked a decrease in tetanic contraction. In the present study, leucine supplementation prevented fatigue in uninjured muscles, which may be due to a greater accumulation of glycogen and an increase in the content of intracellular ATP after supplementation, as it has been shown in gastrocnemius muscles treated with the leucine metabolite beta-hydroxy-beta-methylbutyrate [48]. We also found that leucine treatment prevented fatigue in injured muscles, indicating that the gain in myofiber CSA through leucine-induced attenuation of protein ubiquitination was relevant to muscle function. Alternatively, the mechanism of the leucine-associated increase in myofiber caliber during regeneration might involve an increase in the proliferation of myogenic precursor cells, given that leucine has been shown to increase the *in vitro* proliferation of satellite cells isolated from pigs [49].

Although our previous experiments showed that saline-gavaged rats did not have morphological changes compared to those from intact animals (data not shown), future investigations should use a more appropriate control group by supplementing animals with equimolar doses of some other amino acid besides leucine. This procedure would systematically help to confirm the beneficial effects of leucine on skeletal muscle regeneration.

To our knowledge, this is the first study to demonstrate that leucine supplementation improves the recovery of myofiber size and muscle function of regenerating soleus muscle through attenuation of protein ubiquitination. These findings are relevant for clinical application in an effort to accelerate recovery of function in conditions of muscle injury (e.g. sport injuries, muscle damage caused by surgical intervention and frostbite) when appropriate repair of muscle can significantly decrease rehabilitation time. Further studies are needed in order to investigate the potential therapeutic effects of leucine in muscle disorders involving muscle damage.
